# Biliary Migration, Colonization, and Pathogenesis of *O. viverrini* Co-Infected with *CagA+ Helicobacter pylori*

**DOI:** 10.3390/pathogens10091089

**Published:** 2021-08-26

**Authors:** Watcharapol Suyapoh, Janina E. E. Tirnitz-Parker, Sirikachorn Tangkawattana, Sutas Suttiprapa, Banchob Sripa

**Affiliations:** 1 Biomedical Sciences Program, Graduate School, Khon Kaen University, Khon Kaen 40002, Thailand; watcharapol_su@hotmail.com; 2 WHO Collaborating Centre for Research and Control of Opisthorchiasis (Southeast Asian Liver Fluke Disease), Faculty of Medicine, Khon Kaen University, Khon Kaen 40002, Thailand; sirikach@kku.ac.th (S.T.); sutasu@kku.ac.th (S.S.); 3 Liver Disease and Regeneration Group, Curtin Medical School, Curtin Health Innovation Research Institute, Curtin University, Perth 6102, Australia; n.tirnitz-parker@curtin.edu.au; 4 Faculty of Veterinary Medicine, Khon Kaen University, Khon Kaen 40002, Thailand; 5 Tropical Medicine Graduate Program, Faculty of Medicine, Khon Kaen University, Khon Kaen 40002, Thailand; 6 Tropical Disease Research Center, Department of Pathology, Faculty of Medicine, Khon Kaen University, Khon Kaen 40002, Thailand

**Keywords:** *Opisthorchis viverrini*, *Helicobacter pylori*, co-infection, migration, colonization, pathogenesis

## Abstract

Co-infection with the *cag*A strain of *Helicobacter pylori* exacerbates the pathology of human liver fluke *Opisthorchis viverrini* (OV) infection leading to cholangiocarcinoma. However, underlying mechanisms remain unclear. We report a significant increase in *cag*A-positive and *cag*A-negative *H. pylori* in the stomach, blood, bile, and in the OV worms of co-infected Syrian golden hamsters at one hour, three hours, and one month, post-infection, compared to hamsters infected with either OV or *H. pylori* alone. Except in the worms, *H. pylori* numbers declined at three months post-infection, particularly in the bile fluid of co-infected animals. Both strains of *H. pylori* were immunohistochemically detected in the tegument of the worm, as well as in the bile duct epithelium when co-infected with *O. viverrine,* but not in *H. pylori* infection alone. Interestingly, only the *cag*A-positive strain was detected in the gut of the worm. Co-infection between *cag*A-positive *H. pylori* and *O. viverrini* resulted in a more severe biliary pathology and decreased E-cadherin expression in vivo and in vitro than those of the *cag*A-negative strain. These data suggest that *O. viverrini* acts as a carrier of *cag*A-positive *H. pylori* and co-migrates to the bile ducts, whereas *O. viverrini* facilitates *H. pylori* colonization and enhances the biliary pathogenesis and carcinogenesis.

## 1. Introduction

Chronic infection of humans with the carcinogenic liver fluke, *Opisthorchis viverrini*, is a major public health problem in the Lower Mekong region, including Thailand, Lao People’s Democratic Republic, Cambodia, and Southern Vietnam. Approximately ten million people in these areas are infected with this particular liver fluke [1]. In Thailand, the highest prevalence is reported in the north-eastern part, where about six million people are infected [2,3]. Infection occurs by consuming raw or undercooked freshwater fish containing the infective stage (metacercaria) of the parasite. After ingestion by the host, the metacercaria excyst in the duodenum. The worm then enters the bile duct via the ampulla of Vater before migrating to the intrahepatic bile ducts, where it develops into an adult worm. Chronic bile duct infection causes several hepatobiliary abnormalities, including biliary periductal fibrosis and the lethal bile duct cancer cholangiocarcinoma (CCA). Thus, *O. viverrini* was classified by the International Agency for Research on Cancer (IARC) as a Group 1 biological carcinogen to humans [4]. Interestingly, recent studies have shown that *O. viverrini* co-infected with *Helicobacter pylori* may exacerbate hepatobiliary diseases and orchestrate opisthorchiasis-mediated CCA [5].

*H. pylori*, a rod-shaped bacterium in the genera Proteobacteria, is classified as a Group 1 carcinogen that has been known to cause gastric cancer [4]. *H. pylori* can be divided into two major subpopulations based on the presence or absence of the *cagA* gene, the key virulence factors involving in pathogenesis of the disease, that encodes the CagA protein: *cagA*-positive and *cagA*-negative strains [4]. Proteobacteria are a key population of microbiotas detected in worms, bile samples, and the feces of hamsters infected with *O. viverrini* [6]. A subsequent study has revealed that the liver fluke is a reservoir host of *H. pylori* [7]. Furthermore, co-infection of *O. viverrini* with *H. pylori* enhanced hepatobiliary inflammation and periductal fibrosis in a hamster model [8]. In humans from *O. viverrini-*endemic areas, the *H. pylori* infection rate is higher in *O. viverrini*-infected than uninfected residents [9]. Of note, *H. pylori* bacterial loads are positively correlated with the intensity of *O. viverrini* infection. Interestingly, biliary periductal fibrosis, the major pathologic characteristic of chronic opisthorchiasis, is associated with *cag*A-positive *H. pylori* [9]. Boonyanugomol et al. [10] reported a significantly higher rate of *H. pylori* detection in bile samples of CCA patients (66.7%) compared to non-cancer controls (25.0%). Moreover, *cag*A-positive *H. pylori* had a significantly stronger association with CCA than cholelithiasis or non-cancer controls in the study. These data suggest that *cagA*-positive *H. pylori* is involved in the pathogenesis of hepatobiliary abnormalities and CCA. 

However, despite a strong link between the two carcinogenic pathogens mentioned above, underlying mechanisms remain elusive. Therefore, this study in a hamster model aimed to investigate the relationship between *O. viverrini* and *H. pylori*, with particular focus on how *H. pylori* migrates, colonizes, and induces pathologies of the bile ducts in co-infection with *O. viverrini*. 

## 2. Materials and Methods

### 2.1. Metacercaria Preparation

*O. viverrini* infective stage metacercariae were obtained from naturally infected freshwater cyprinoid fish in an endemic area of Thailand. Five kg of fresh fish were minced and digested in synthetic stomach juice containing 0.15% hydrochloric acid (HCl) and 0.25% pepsin and incubated in a water bath at 37 °C for 1 h. After digestion, the suspension was filtered in a series of copper sieves with different pore sizes to remove solid particles and sedimented in normal saline, as reported previously [11]. *O. viverrini* metacercariae were identified under a dissecting microscope as previously described [12]. Fifty metacercariae were fed to the hamsters via intragastric intubation.

### 2.2. Bacterial Preparation

Two bacterial strains, *cag*A-positive and *cag*A-negative *H. pylori* tagged with green fluorescent protein mutant 2 gene (*gfp*mut2) were kindly provided by Prof. R. Haas, Max von Pettenkofer Institut für Hygiene und Medizinische Mikrobiologie [13]. Both strains were grown on GC selective agar plates, supplemented with 6 mg/L chloramphenicol (chloramphenicol supplement, Oxoid Limited, Hampshire, England), 10mL/L vitox (Vitox supplement, Oxoid Limited, Hampshire, England), Dent (*H. pylori* selective supplement, Oxoid Limited, Hampshire, England), and horse serum (5%). The plates were incubated for 4–5 days at 37 °C in 6–12% O_2_ and 5–8% CO_2_ microaerobic atmosphere adjusted by 1 sachet/2.5L jar of gas generator (AnaeroPack-MicroAero Gas Generator, Mitsubishi Gas Chemical Co. Ltd., Tokyo, Japan) [14]. After sufficient bacterial growth, *H. pylori* colonies were suspended in *Brucella* broth, mixed, and washed several times, and adjusted to 1.7 × 10^8^ colony forming units (CFUs)/mL [15]. Hamsters were given 1 mL of the bacterial solution orally, using a stomach tube 24 h after fasting.

### 2.3. Experimental Design

**Figure 1:**
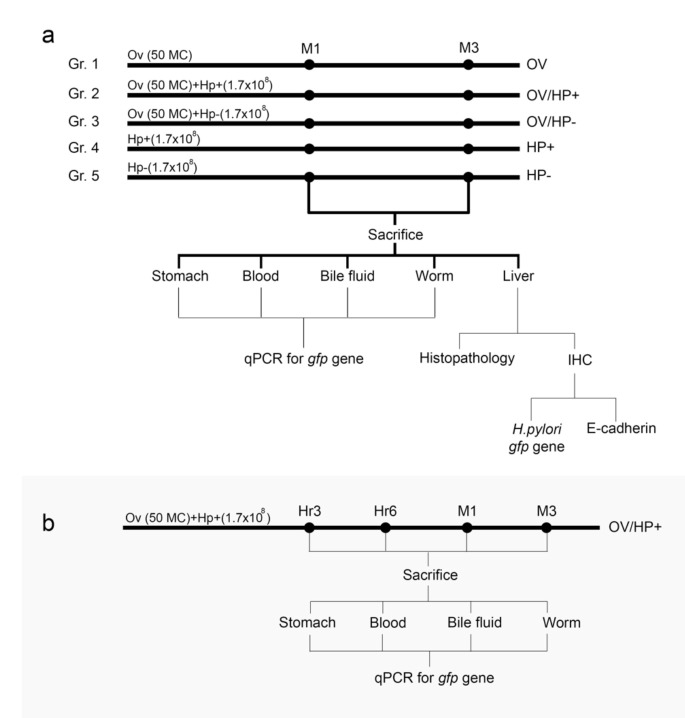
Experimental design. (**a**). Long-term study using 5 different infection groups of hamsters sacrificed, and sample collected at 1 month-6 months. (**b**). Short-term study using *O. viverrini* and *cag*A-positive *H. pylori* co-infected and sample collected at 1 and 3 h, 1 month, and 3 months. OV = *O. viverrini* infection alone, OV/HP- = *O. viverrini* infection + *cag*A-negative *H. pylori*, OV/HP+ = *O. viverrini* infection + *cag*A-positive *H. pylori*, HP+ = *cag*A-positive *H. pylori* infection alone, HP- = *cag*A-negative *H. pylori* infection alone.

Six-week-old male Syrian hamsters (n = 50) were assigned to 5 groups of 10 animals each (Figure 1a). Group I (OV): *O. viverrini* infection, Group II (OV/HP+): *O. viverrini* + *cag*A-positive *H. pylori* infection, Group III (OV/HP-): *O. viverrini* + *cag*A-negative *H. pylori* infection, Group IV (HP+): *cag*A-positive *H. pylori* infection, Group V (HP-): *cag*A-negative *H. pylori* infection. All animals were kept under controlled temperature and day/night cycle conditions and provided with food and water *ad libitum.* This animal study was approved by the Animal Ethics Committee of Khon Kaen University (AEKKU #41/2561).

Five hamsters per group were euthanized by isoflurane inhalation at 1 and 3 months after infection and the liver, stomach, bile fluid, blood, and worms were collected. The liver was fixed in 10% neutral buffered formalin and processed by routine paraffin histological technique. The paraffin sections were then used for immunofluorescence and immunohistochemistry studies.

Additional experiments for a short-term study were *O. viverrini* and *cag*A-positive *H. pylori* co-migration assigned at 1 and 3 h after infection (Figure 1b). Five hamsters per time period were euthanized and samples were collected as outlined above. 

### 2.4. DNA Extraction

Whole blood (250 μL per animal) was lysed repeatedly in red blood cell lysis buffer (0.32 M sucrose, 10 mM Tris HCL, 5 mM MgCl_2_, 0.75% Triton-X-100) until white pellet was obtained [16]. Whole bile fluid was centrifuged at 5000× g for 5 min at 4 °C. Blood and bile pellets, 1 g of stomach tissue and whole worm samples were used for DNA extraction using the standard phenol-chloroform method [17]. Briefly, the samples were incubated with a DNA extraction buffer consisting of 20 mM Tris-HCl, 1 mM EDTA, 10 mM NaCl, 10% sodium dodecyl sulfate, adjusted to pH 8. The stomach and worm samples were homogenized with a tissue grinder and digested with proteinase K (10 mg/mL of proteinase K, 50 mM Tris-HCl, adjusted to pH 7.5) at 55 °C overnight. The DNA was then separated from the sample solutions by phenol and chloroform extraction and precipitated with ethanol. The DNA concentration was measured by using a NanoDrop® ND-1000 UV-Vis Spectrophotometer (Thermo Fisher Scientific, Inc., Waltham, MA, USA) and adjusted to 50 ng/μL.

### 2.5. Real-Time Polymerase Chain Reaction (qPCR) for gfp Gene Detection

Recombinant plasmid, phel12 containing *gfp* was extracted using the Plasmid Extraction kit (GeneJET Plasmid Miniprep Kit, Thermo Fisher Scientific, Inc., Waltham, MA, USA) and used to prepare a qPCR standard curve. The DNA copy number was calculated as previously published [18] with a size of phel12 containing *gfp* of 5116 bp. The plasmid solution was serially diluted 10-fold, resulting in dilutions ranging from 1 ng/μL to 1 fg/μL. Absolute quantification was performed by generating a standard curve for each *gfp* and plotting the quantification cycle (Cq) values against log [quantity] of a dilution series of known *gfp* amounts.

The reaction mixture consisted of 10 μL of 1x Master Mix (FastStart Universal SYBR Green Master (Rox), Roche, Mannheim, Germany) containing FastStart Taq DNA polymerase, reaction buffer, nucleotides (dATP, dCTP, dGTP, and dTTP), SYBR Green I and a reference dye. A *gfp* primer set, forward- TCCATGGCCAACACTTGTCA and reverse- CATAACCTTCGGGCATGGCA in a volume of 0.6 μL of 300 nM were added to the reaction tube, with 6.8 μL of sterile distilled water mixed with diethylpyrocarbonate (DEPC). The DNA sample was used at 2 μL per reaction tube, making a final reaction volume of 20 μL. The PCR and melting curve conditions were set as 94 °C 30 s, 58 °C 30 s, 72 °C 45 s (50 cycles) and 95 °C 30 s, 60 °C 60 s, 95 °C 15 s. Expected gene product size was 112 bp.

### 2.6. Immunofluorescence for Localization of H. pylori-GFP

The liver tissue sections were deparaffinized and rehydrated prior to antigen retrieval by microwaving in 10 mM citric acid, pH 6.0 at 100 W for 5 min and 20 W for 15 min. The sections were cooled down for 30 minutes, washed twice in phosphate-buffered saline (PBS), and endogenous peroxidases blocked with 3% H_2_O_2_ in methanol. After blocking non-specific protein binding with 1% bovine serum albumin for 1 h, the sections were incubated with mouse anti-GFP monoclonal antibody (Clone: GFP-20; G6539, Sigma-Aldrich, St. Louis, Missouri, MO, USA) at a dilution of 1:1000 in Tris-buffered saline (TBS) at 4 °C overnight. After washing, the sections were incubated with the secondary antibody (1:500 dilution), Alexa488-labeled goat-anti-mouse immunoglobulin (Thermo Fisher Scientific, Inc., Waltham, MA, USA) for 30 min. After staining with Hoechst dye (1:2000 dilution in TBS) for 10 min, the stained sections were mounted with 10% glycerol and observed under a fluorescence microscope (Olympus BX51, Olympus Corporation, Shinjuku-ku, Tokyo, Japan). The expression of GFP was confirmed by double-immunofluorescence staining with 1:50 in TBS rabbit anti-*H. pylori* polyclonal primary antibody (*H. pylori* strain CH-20426; B0471, DakoCytomation, Glostrup, Denmark). Secondary antibodies used were goat anti-Mouse Alexa488 (1:500 dilution in TBS) and donkey anti-rabbit Alexa594 (1:500 dilution in TBS) (Thermo Fisher Scientific, Inc., Waltham, MA, USA) for 1 h. 

### 2.7. O. viverrini Excretory-Secretory Products Preparation

Excretory-secretory products were obtained by culturing adult worms in vitro at 37 °C in RPMI-1640 containing penicillin (100 U/mL), streptomycin (100 μg/mL), and protease inhibitors (0.1 mM phenylmethanesulfonyl fluoride (PMSF), 1 mM leupeptin and 0.1 mM N-[N-(L-3-trans-carboxyoxiran-2-carbonyl)-L- leucine]-agmatine, E-64). Excretory-secretory products was collected every 12 h for 7 days from the medium of live worms. The medium was centrifuged to remove the eggs (1000× *g*, 10 min at 4 °C). The clear supernatant was concentrated, and lipopolysaccharide (LPS) removed with Triton-X114 (Sigma-Aldrich, Missouri, MO, USA) as previous described [19]. After removal of residual Triton-X114 by Bio-Beads SM2 (Bio-Rad, California, CA, USA), the excretory-secretory products were filtered through a 0.2 micrometer membrane, protein measured, aliquoted and stored at −80 °C for subsequent in vitro assays.

### 2.8. H. pylori Adhesion Assay

A normal biliary epithelial cell line H69 was seeded (1 × 10^5^ cells/culture) on NaOH-treated cover slides and cultured in enriched medium, which was a mixture of 45% Dulbecco’s Modified Eagle’s Medium: Nutrient Mixture F-12 (DMEM/F12, Gibco/ Thermo Fisher Scientific, Inc., Waltham, MA, USA), 45% DMEM with high glucose (Gibco/ Thermo Fisher Scientific, Inc., Waltham, MA, USA) supplemented with 10% fetal bovine serum, penicillin (100 U/mL), streptomycin (100 U/mL), adenine 25 µg/mL, insulin 5 µg/mL, epinephrine 1 µg/mL, hydrocortisone 0.62 µg/mL, T3 13.6 ng/mL, epidermal growth factor 10 ng/mL and incubated at 37 °C in a 5% CO_2_ humidified atmosphere for three days. The H69 cells were either treated with *H. pylori* at 80% confluency at 1:1 multiplicity of infection (MOI) or exposed to 1:1 MOI *H. pylori* under the presence of 20 µl/mL of excretory-secretory products for 24 h. After incubation, the cells were washed with sterile PBS and fixed in 1:1 methanol: acetone for 5 min. After washing, the cells were incubated with DAKO protein blocking solution (Dako Protein Block Serum Free: X0909, DakoCytomation, Glostrup, Denmark) for 20 min at room temperature and incubated with 1:500 dilution of mouse anti-*H. pylori* monoclonal antibody (Clone: BD1586; SC57780; Santacruz Biotechnology, Texas, Vt., USA) and rabbit anti-beta actin polyclonal primary antibody (1:250 dilution) (Ab8227; Abcam, Cambridge, United Kingdom) for 1 h. The cells were then washed and incubated with 1:500 goat anti-mouse IgG-Alexa488 and 1:500 donkey anti-Rabbit IgG-Alexa594 (Thermo Fisher Scientific, Inc., Waltham, MA, USA) for 1 h. After thorough washes with PBS, the cells were mounted with DAPI medium (ProLong Gold antifade reagent, Thermo Fisher Scientific, Inc., Waltham, MA, USA) for nuclear visualization and viewed under a fluorescence microscope (Olympus BX51, Olympus Coperation, Shinjuku-ku, Tokyo, Japan). The number of adherent *H. pylori* was manually evaluated at 40× magnification for 10 non-overlapping fields of view. 

### 2.9. Epithelial Transmigration Assay

The epithelial transmigration of *H. pylori* was studied using the H69 cell line with the number of cells and slide preparation performed as described for the *H. pylori* adhesion assay. For the experimental design, the cell lines were assigned to two groups. In group 1, *cag*A-positive *H. pylori* was added to 80% confluent cell cultures at 1:1 MOI. For group 2, the 80% confluent cultures were exposed to 1:1 MOI *cag*A-positive *H. pylori* under the presence of 20 μg /mL of excretory-secretory products. Both groups were cultured for 3, 6 and 24 h. After incubation, the cells were washed with sterile PBS and fixed in 1:1 methanol: acetone for 5 min. The cells were then incubated with DAKO protein blocking solution (Dako Protein Block Serum Free: X0909, DakoCytomation, Glostrup, Denmark) for 20 min at room temperature. The cell culture slides were incubated with primary antibodies 1:500 dilution of mouse anti-*H. pylori* monoclonal primary antibody (Clone: BD1586; SC57780; Santacruz Biotechnology, Texas, Vt., USA) in TBS and rabbit anti-E-cadherin (24E10) polyclonal primary antibody (1:200 dilution in TBS) (3195; Cell Signaling, Massachusetts, Mass., USA) for 1 h, washed, and then incubated with secondary antibodies 1:500 goat anti-mouse Alexa488 and 1:500 donkey anti-rabbit Alexa594 (Thermo Fisher Scientific, Inc., Waltham, MA, USA) for 1 h. The cells were mounted with DAPI medium (ProLong Gold antifade reagent, Thermo Fisher Scientific, Inc., Waltham, MA, USA) and evaluated for evidence of *H. pylori* transmigration across the cell cleft at 40–100× magnification under a confocal microscope. The multi-image layers were generated on the UltraVIEW VoX Spinning Disk confocal microscope from PerkinElmer using Volocity software while the 3D construction pictures were performed by confocal scanning microscope LSM800 from Carl Zeiss using Zen black software.

### 2.10. Histopathology and E-cadherin Immunohistochemistry

Liver tissue sections were deparaffinized and rehydrated before staining with hematoxylin and eosin (H&E) (Sigma-Aldrich, Missouri, MO, USA). Histopathology such as periductal inflammation, goblet cell metaplasia, biliary dysplasia and mitotic figures were investigated. 

E-cadherin expression was detected by immunohistochemistry similar to the immunofluorescence staining mentioned above. Briefly, liver sections were incubated with anti-E-cadherin monoclonal primary antibody (Clone: NCH-38; M3612; DakoCytomation, Glostrup, Denmark) at 1:50 dilution in TBS at 4 °C overnight. The slides were then incubated with biotinylated goat antibody mouse/rabbit immunoglobulin (K0675; DakoCytomation, Glostrup, Denmark) in TBS at 1:100 dilution for 1 h. After three thorough washes, the slides were incubated in streptavidin-biotin-horseradish peroxidase (HRP) complex (Enzyme Label) (K0355; DakoCytomation, Glostrup, Denmark) solution for 1 h. Unbound excess streptavidin complexes were removed through thorough washes and the slides were then developed in diaminobenzidine tetrahydrochloride solution for 5 min and rinsed in tap water. The liver sections were counterstained in Mayer’s hematoxylin, dehydrated, cleared, mounted and observed under a light microscope. Positive biliary E-cadherin expression, as identified by brown staining, was quantified in the first and second order bile ducts in 10 non-overlapping fields of view at 20× high magnification (Olympus BX51, Olympus Coperation, Shinjuku-ku, Tokyo, Japan) using ImageJ software [20]. 

### 2.11. Data Analysis

All data were analyzed using SPSS version 23.0 (SPSS Inc., Chicago, IL, USA). The t-test was used to compare means between two groups and the analysis of variance (ANOVA) with post-hoc (LSD) was used to compare multiple groups. A *p* value of < 0.05 and < 0.01 were considered as statistically significant.

## 3. Results

### 3.1. Organ Distribution of H. pylori in Infected Hamsters

**Figure 2:**
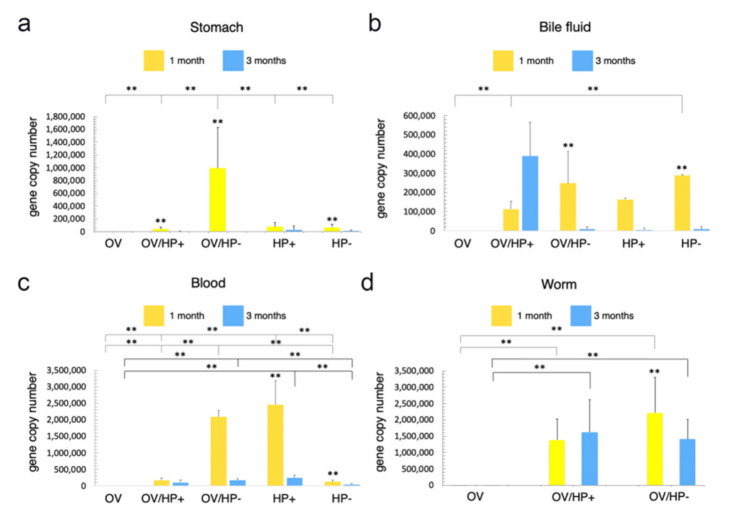
*gfp* copy numbers representing the presence of *H. pylori* in the stomach (**a**), bile fluid (**b**), blood (**c**) and worms (**d**) from five different infection groups at 1- and 3-month post-infection. Statistical test using ANOVA, ** *p* < 0.01. OV = *O. viverrini* infection alone, OV/HP- = *O. viverrini* infection + *cag*A-negative *H. pylori*, OV/HP+ = *O. viverrini* infection + *cag*A-positive *H. pylori*, HP+ = *cag*A-positive *H. pylori* infection alone, HP- = *cag*A-negative *H. pylori* infection alone.

To investigate the route of *H. pylori* migration to the bile duct, we first explored the *H. pylori* distribution in the relevant tissues. We used qPCR to quantify the expression of *H. pylori-*associated *gfp* gene through copy number analysis of both transfected *H. pylori* strains in the stomach, blood, and bile fluid of each animal from all experimental groups, at one and three months after infection. In the *O. viverrini* co-infection group, the number of *H. pylori* in the worm was also examined. At 1 month post-infection, *cag*A-negative *H. pylori* was detected at significantly higher levels in the gastric mucosa of *O. viverrini* co-infected hamsters than in animals infected with *cag*A-positive, or *cag*A-negative or *cag*A-positive alone (Figure 2a). As expected, the *H. pylori* copy numbers of both strains in the stomach were significantly reduced at 3 month post-infection in all experimental groups. Both *H. pylori* strains were detected in the bile fluid and blood samples, regardless of *O. viverrini* co-infection. However, the copy numbers were significantly reduced 3-months post-infection (Figure 2b–d). Interestingly, significantly higher *cag*A-positive *H. pylori* copy numbers were observed in the bile fluid samples at 3 months post-infection in the *O. viverrini* infection group, compared to animals co-infected with *cag*A-negative, or *cag*A-positive or *cag*A-negative *H. pylori* alone (Figure 2b). Both *cag*A-positive and *cag*A-negative *H. pylori* were found in the worms of the *O. viverrini* infection groups. However, the *cag*A-negative *H. pylori* copies were significantly decreased at 3 months compared to 1 month post-infection, whereas the *cag*A-positive counterparts showed slightly increased levels at 3 month post-infection (Figure 2d). No *gfp*-transfected *H. pylori* was found in any samples of *O. viverrini* infection alone (control) (Figure 2a–d). Detailed comparisons including the statistical analyses are described in the figure legends.

### 3.2. Route of Migration of H. pylori in O. viverrini Co-Infection

**Figure 3:**
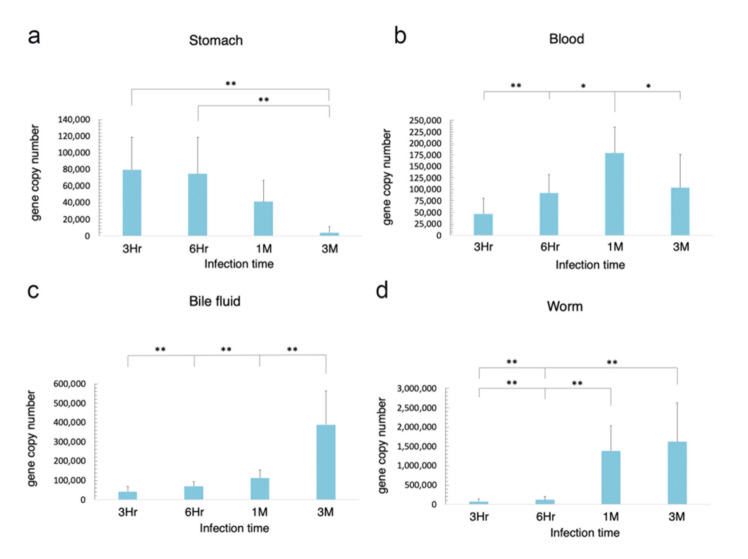
*H. pylori* copies based on *gfp* detection in the stomach (**a**), blood (**b**), bile fluid (**c**) and worms (**d**) of *O. viverrini* co-infected with *cag*A-positive *H. pylori* at 3 h, 6 h, 1 month and 3 months post-infection. Statistical test using ANOVA, * *p* < 0.05, ** *p* < 0.01. Hr = hour, M = month.

The results from the distribution study led us to analyse the route of migration of *cag*A-positive *H. pylori* co-infection with *O. viverrini* for further investigation. The animals were co-infected with *cag*A-positive *H. pylori* and *O. viverrini* and examined for *gfp* copies (representing *H. pylori*) in the stomach, blood, bile fluid, and worms at 3 h, 6 h, 1 month, and 3 months post-infection. *H. pylori*, as detected by *gfp* gene quantification, was observed at 3 h post-infection through to the end of experiments in all examined tissues, including the worms themselves (Figure 3a–d). The *H. pylori* levels in the stomach were gradually reduced from 3 h to 1 month and significantly decreased at 3 months post-infection (Figure 3a), whereas those in the bile fluid and worms were significantly increased at 1 month and 3 months post-infection (Figure 3c,d). The *H. pylori* levels in the blood were gradually increased and reached their maximum at 1 month, before levels decreased again and were significantly reduced at 3 months post-infection compared to the first month (Figure 3b). Details of the statistical analyses are described in the figure legends.

### 3.3. O. viverrini Enhances H. pylori Colonization In Vivo

**Figure 4:**
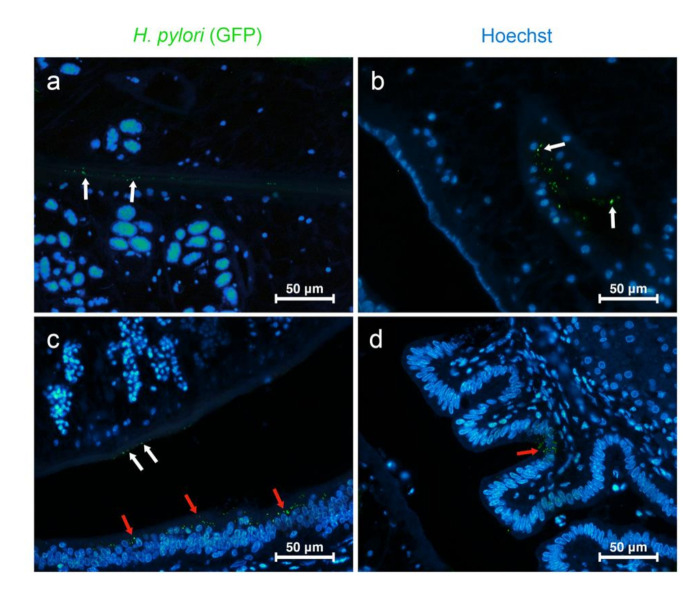
Representative pictures of *H. pylori* colonization. (**a**) *H. pylori* on the peri-tegumental surface of *O. viverrini* (white arrow); (**b**) *H. pylori* in the gut lumen and epithelium (White arrow); (**c**) *H. pylori* at the worm tegument (white arrow) and the 1st order bile duct epithelial cells (red arrow); (**d**) *H. pylori* in the epithelial crypt. The green channel represents GFP detected by Alexa488-labeled antibody. Blue channel represents cell nuclei by Hoechst staining. [**a**–**d** = Immunofluorescence, original magnification, (**a**–**c**) ×40, (**d**) ×60, scale bar depicts 50 µm].

**Figure 5:**
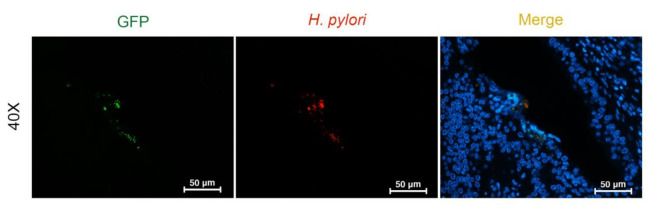
Co-expression of GFP and *H. pylori* in the biliary epithelium using immunofluorescent staining. The green channel represents GFP visualised with the Alexa488 antibody. The red channel represents *H. pylori* by the Alexa594 antibody. The blue channel represents nuclei by Hoechst staining. Merged yellow spots identifies the co-localization of both GFP and *H. pylori* (Immunofluorescence, original magnification, ×40, scale bar depicts 50 µm).

Next, we investigated the effect of *O. viverrini* on *H. pylori* colonization in the bile ducts using a well-studied hamster model. Paraffin-embedded infected liver tissues were used to examine the presence of *H. pylori*. The number of *H. pylori* in the bile ducts and in the worms themselves were assessed semi-quantitatively by using immunofluorescent staining for green fluorescent protein (GFP). Both strains of *H. pylori* were observed at the biliary epithelial cell surface and perinuclear area in *O. viverrini* co-infected with *H. pylori,* but not in *H. pylori* infection alone (Table 1 and Figure 4a–d). In addition, only *cag*A-positive *H. pylori* were also detected in the crypt of the large bile duct epithelium (Figure 4d). No *H. pylori* were detected in the small bile ducts. Both *H. pylori* strains were found on the tegument of the *O. viverrini* worms. However, only the *cag*A-positive strain was observed in the worm’s gut (Table 1 and Figure 4b). The presence of GFP in *H. pylori* was confirmed by double immunofluorescence using antibodies to GFP and *H. pylori* (Figure 5).

### 3.4. O. viverrini Excretory-Secretory Products Enhance H. pylori Binding to Bile Duct Epithelial Cells In Vitro 

**Figure 6:**
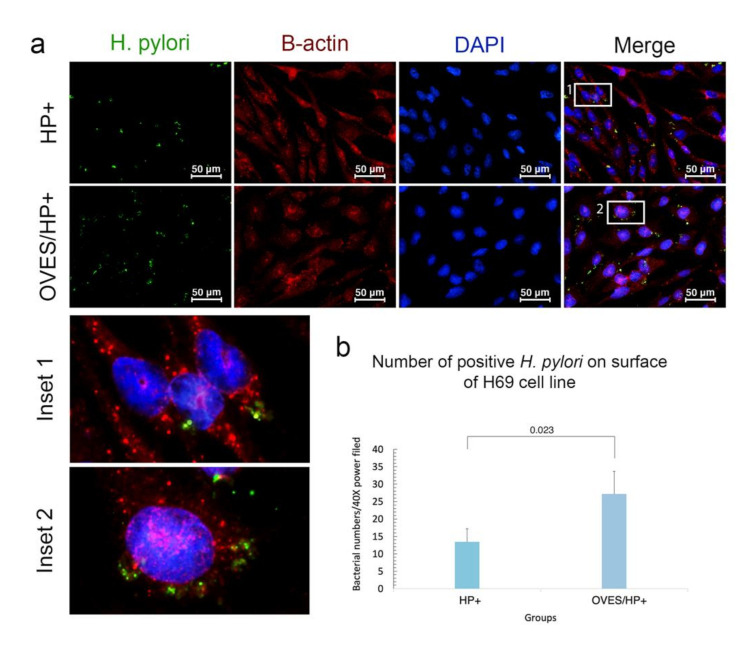
Adhesion assay of *H. pylori* to the biliary epithelial cell line. (**a**), representative pictures depict different amounts of *H. pylori* on the H69 cell surface, HP+ = *H. pylori cag*A*+* bacteria alone, OVES/HP+ = excretory-secretory products + *H. pylori cag*A+ bacteria. (**b**), significant bacterial number on H69 cells with excretory-secretory products than those without (*p* = 0.023). The green channel represents *H. pylori* by the Alexa488-labeled anti-*H. pylori* antibody. Red channel represents β-actin of H69 by the Alexa594-labeled anti-β-actin antibody. Blue channel represents the nuclei by DAPI staining [Immunofluorescence, original magnification, ×40, scale bar depicts 50 µm].

To explore the effects of *O. viverrini* on *H. pylori* colonization, we performed an in vitro binding assay using the cholangiocyte cell line, H69 cocultured with *cag*A-positive *H. pylori* with or without *O. viverrini* excretory-secretory products. Fluorescent bacterial binding to H69 cells was assessed and counted per high power field (HP). The results showed that *H. pylori* adhered to the cell surface but was not detected in the cytoplasm (Figure 6a). Quantitatively, *O. viverrini* excretory-secretory products significantly enhanced the binding of *H. pylori* to the bile duct cell line (27 ± 6.5 cells/HP) compared to those incubated with *H. pylori* alone (13 ± 3.8 cells/HP) (*p* = 0.023) (Figure 6b).

### 3.5. Effect of O. viverrini Excretory-Secretory Products on H. pylori Trans-Epithelial Migration and E-Cadherin Expression In Vitro

**Figure 7:**
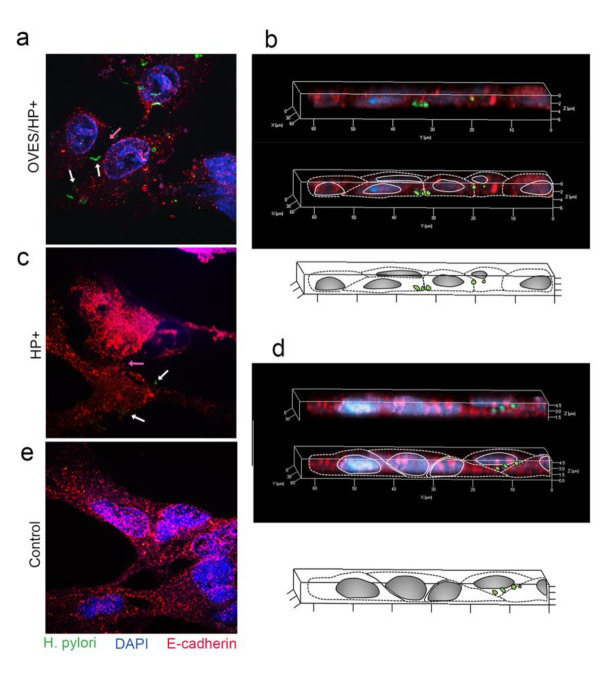
Confocal photomicrograph representing *H. pylori* on the epithelial gap. (**a**,**b**); OVES/HP+ = cell treated with excretory-secretory products + *cag*A-positive *H. pylori*. (**c**,**d**); HP+ = cell treated with *cag*A-positive *H. pylori*. (**a**,**c**); the sectioned photomicrograph represents E-cadherin junction and transmigrated *H. pylori*. (the pink arrow points towards the epithelial gap, the white arrow identifies *H. pylori*) (**b**,**d**); 3D-reconstruction image of both co-incubation groups. The top picture is a 3D-reconstruction photomicrograph. The middle picture is a photomicrograph with an overlay of the diagram outlined below. (**e**); control H69 cells without OVES or HP+ co-incubation. (Original magnification, ×100, scale bar depicts 5 µm).

To determine the effect of *O. viverrini* excretory-secretory products on *H. pylori,* transmigration, immunofluorescent staining and confocal microscopy were utilized to determine the location of *H. pylori* and the expression of E-cadherin in H69 biliary cells after 3, 6, and 24 h co-incubation with and without excretory-secretory products at 3 and 6 h, the bacteria in excretory-secretory products co-incubated (OVES/HP+) and HP+ alone (HP+) were observed on the H69 apical surface, but not in the intercellular space. Interestingly, at 24 h, *H. pylori* was detected at the basolateral surface in the OVES/HP+ biliary cells (Figure 7a) but not in cells incubated with HP+ alone (Figure 7c). Three-dimensional reconstruction images confirmed the presence of *H. pylori* at the basolateral surface of the biliary cells co-incubated with excretory-secretory products (Figure 7b) but not HP+ alone (Figure 7d). Concurrently, the level of E-cadherin expression was reduced in OVES/HP+ (Figure 7a) compared to those of HP+ alone (Figure 7c). Control cells with no HP+ and OVES showed normal E-cadherin expression with no *H. pylori* detection (Figure 7e).

### 3.6. Co-Infection of cagA-Positive H. pylori and O. viverrini Enhances Biliary Epithelial Pathological Changes

**Figure 8:**
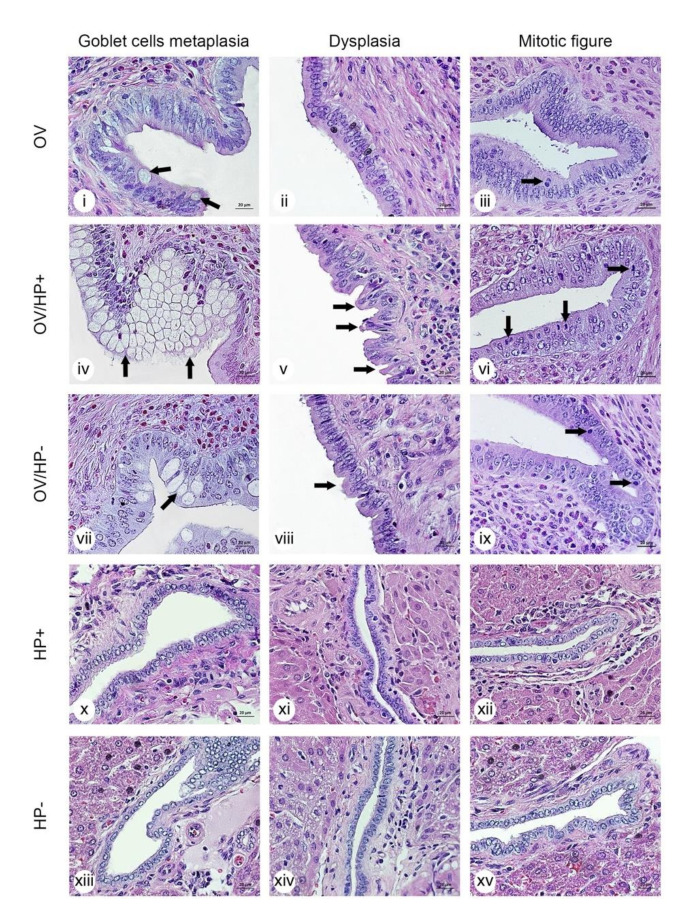
Representative micrographs of histopathological lesions at 3 months post-infection in 5-different experimental groups. OV = *O. viverrini* infection alone, OV/HP- = *O. viverrini* infection + *cag*A-negative *H. pylori*, OV/HP+ = *O. viverrini* infection + *cag*A-positive *H. pylori*, HP+ = *cag*A-positive *H. pylori* infection alone, HP- = *cag*A-negative *H. pylori* infection alone. Micrographs of goblet cell metaplasia in OV, OV/HP+, OV/HP- (**i**, **iv**, **vii**) and no metaplasia development in HP+, HP- (**x**, **xiii**), biliary dysplasia in OV/HP+, OV/HP- (**v**, **viii**) and no dysplasia development in OV, HP+, HP- (**ii**, **xi**, **xiv**), and proliferation (mitotic figures are identified with a black arrow) of OV, OV/HP+, OV/HP- (**iii**, **vi**, **ix**) and no proliferation in HP+, HP-, respectively (**xii**, **xv**). Micrographs in rows represent lesions in each treatment group. H&E staining, original magnification, ×40.

Based on the hypothesis that *O. viverrini* facilitates *H. pylori* migration and colonization, we further investigated the potential enhancement role of *H. pylori* in the pathogenesis of opisthorchiasis and its associated CCA in a hamster model. Biliary changes including goblet cell metaplasia, biliary dysplasia and biliary proliferation were assessed by histopathology at 1- and 3-months post-infection. Overall, the epithelial changes were limited to the first order bile ducts the liver fluke inhabited (Figure 8i–ix). These included goblet cell metaplasia, biliary dysplasia, and proliferation. At 1-month post-infection, the lesions showed less severe pathological changes compared to the lesions from animals infected for 3 months. The pathological changes in the bile duct lesions were significantly progressed 3 months post-infection (Figure 8). *O. viverrini* infection induced goblet cell metaplasia (Figure 8i), biliary dysplasia (Figure 8ii) and proliferation (demonstrated by mitotic figures) (Figure 8iii) of the bile duct epithelia. Co-infection with *cag*A-positive *H. pylori* induced more severe goblet cell metaplasia, dysplasia, and cell proliferation (Figure 8iv,v,vi). No such enhancement was observed in *cag*A-negative *H. pylori* co-infected with *O. viverrini* hamsters (Figure 8vii,viii,ix). None of these pathological changes were seen in *H. pylori* infection alone (Figure 8x–xv). 

### 3.7. Co-Infection of cagA Positive H. pylori and O. viverrini Reduces E-Cadherin Expression In Vivo

**Figure 9:**
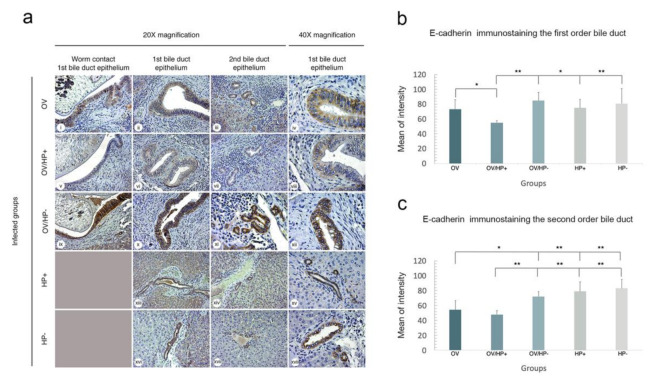
Immunohistochemical staining of E-cadherin in bile duct epithelia in each experimental group. (**a**) representative micrographs of E-cadherin staining in each group. Column 1, 2, and 4 represented the 1st order bile duct, Column 3 represented 2nd bile duct. The expression of E-cadherin was detected strongest colour in OV/HP- (**ix**–**xii**), HP+ (**xiii**–**xv**), and HP- (**xvi**–**xviii**). Moderate colour in OV (**i**–**iv**), weakest colour in OV/HP+ (**v**–**viii**). (**b**) the staining intensity was compared by means of positively stained biliary cell numbers in the first-order bile duct. (**c**) in the second-order bile duct. Significant differences are shown as * *p* < 0.05, ** *p* < 0.01. (Column 1–3 = original magnification, ×20; Column 4 = original magnification, ×40).

Lastly, to assess the carcinogenic role of *H. pylori/O. viverrini* co-infection, expression of E-cadherin as a key cell adhesion molecule generally associated with malignant transformation-associated processes, was investigated in the biliary epithelium of infected hamsters. Qualitatively, intense cytoplasmic E-cadherin expression was observed in both the first and second order bile ducts of uninfected control hamsters. The expression of E-cadherin was quantitatively scored as mean intensity using ImageJ software. The results showed that E-cadherin expression levels in the bile duct epithelial cells was significantly lower in *O. viverrini-*infected groups (OV, OV/HP+, OV/HP-) compared to *H. pylori-*infected groups (HP+, HP-) (Figure 9a–c). Importantly, co-infection of *cag*A-positive *H. pylori* and *O. viverrini* significantly reduced the E-cadherin expression (Figure 9v–viii). In contrast, *cag*A-negative *H. pylori* co-infected with *O. viverrini* had no effect on E-cadherin levels (Figure 9ix–xii), similar to data obtained with *H. pylori* infection alone (Figure 9xiii–xviii). Significant down-regulation of E-cadherin was found in both first and second order bile ducts of *cag*A-positive *H. pylori* co-infected with *O. viverrini* (Figure 9b,c).

## 4. Discussion

Liver fluke infection caused by *O. viverrini* is a major public health problem in Southeast Asia and often results in diverse hepatobiliary pathologies including CCA [21]. Our group was the first to describe the association between CCA and *H. pylori*, particularly *cag*A-positive infection in liver fluke endemic areas in Thailand [10]. However, the mechanistic link between *O. viverrini* and *H. pylori* was unclear until recently. We discovered that *O. viverrini* is a reservoir of *H. pylori* [7] and co-infection of the two pathogens can enhance hepatobiliary abnormalities, specifically advanced periductal fibrosis in opisthorchiasis and *cag*A-positive *H. pylori* [9]. However, the mechanisms underlying enhanced carcinogenesis have remained elusive. Here, we showed that *O. viverrini* facilitated *H. pylori* migration, adhesion, and colonization, especially the *cag*A-positive strain. Moreover, co-infection also induced more severe biliary pathology, with down-regulated E-cadherin expression representing potentially malignant transformation. These results significantly contribute to our understanding of the underlying mechanisms and help to clarify how the liver fluke enhances *cag*A-positive *H. pylori-*induced severe opisthorchiasis and CCA. 

*H. pylori*, a gram-negative bacterium, colonizes the gastric mucosa of humans and induces several gastrointestinal disorders, such as chronic gastritis, peptic ulcer, gastric cancer, and mucosa-associated lymphoid tissue lymphoma (MALT) [22,23]. In addition to the stomach, it has been found in the cardiovascular, nervous, pancreatic, and hepatobiliary systems [24,25]. However, the mechanisms by which *H. pylori* migrate to the extra-gastric tissues remain unclear. Two possible routes have been proposed for the migration to the hepatobiliary system: (1) ascending infection and (2) hematogenous spread [26,27,28,29]. In this study, we explored another possible route of *H. pylori* migration to the bile ducts when co-infected with the liver fluke, *O. viverrini*. We were able to detect *H. pylori* in the tegument and gut of the worms and in bile fluids, as early as three hours after infection, which is consistent with the time required for normal migration of *O. viverrini* to the bile ducts [12]. This suggests that *H. pylori* is “piggybacked” and co-migrates to the bile ducts via the juvenile worms. Detection of *H. pylori* in the blood also suggests the hematogenous migration route. However, where, and how *H. pylori* enter the blood circulation remains unknown. Ascending infection via bile duct obstruction is unlikely as no severe periductal fibrosis develops in this early stage. Nonetheless, enhancement of *H. pylori* migration into the biliary system may occur during chronic liver fluke infection. 

Preference of *cag*A-positive *H. pylori* strain colonization in the biliary system in opisthorchiasis was evidenced in this study. By comparing the distribution of *cag*A-positive and *cag*A-negative *H. pylori* in the stomach, blood, bile fluid and adult worms of *O. viverrini*-infected hamsters, we demonstrated that the low virulence strain (*cag*A-negative) easily propagates at early infection (one month) but was significantly reduced at three months post-infection in all locations and worms studied. *O. viverrini* seems to enhance the colonization of the *cag*A-negative *H. pylori* at early stage of infection (one month). However, the mechanism of the rapid reduction of *cag*A-negative strain in chronic infection is unknown. Interestingly, in this study, the significantly higher number of *cag*A-positive compared to the *cag*A-negative *H. pylori* in the bile fluid and the adult worms at chronic stage (three months) implicates the establishment of the bacteria in the biliary system in *O. viverrini* infection. These findings support the significantly higher rates of *cag*A-positive *H. pylori* in opisthorchiasis compared to controls without *O. viverrini* infection in humans [9]. Moreover, the presence and specific hepatic genotypes of the *H. pylori cag*A gene are associated with the pathology of chronic opisthorchiasis, specifically periductal fibrosis [9]. The genetic differences of the gastric and enterohepatic Helicobacter species, reflecting mainly distinct metabolic functions, suggest the evolution and adaptation to different hosts, colonization niches, and mechanisms of virulence [30].

For colonization of *H. pylori* to the bile duct epithelium, we found that *H. pylori* was detected only in the *O. viverrini* co-infection groups. In addition, *H. pylori* colonization was observed only in the first-order bile ducts, where the liver fluke is found, but not in the secondary bile ducts, which are inaccessible to the worms. These results indicate that colonization of *H. pylori* to the bile duct epithelium is *O. viverrini*-dependent in this study. This conclusion is supported by our in vitro experiments, which showed that *O. viverrini* excretory-secretory products increased the binding of *H. pylori* on H69 biliary epithelial cells. Moreover, only *cag*A-positive *H. pylori* was found at the basolateral surface of the biliary cells. This indicates the pathogenetic significance of *cag*A in biliary pathology given that the *cag*A-positive, not the *cag*A-negative *H. pylori* strain is able to colonize the basolateral or intercellular spaces in gastric mucosa of gastritis patients [31]. However, the mechanisms underlying this enhanced colonization phenomenon are unknown. *H. pylori* employs multi-step processes to colonize the gastrointestinal mucosa, including the destruction of the mucous layer and binding to specific host receptors [32]. Several ligands and receptors for *H. pylori* binding have been identified; for example, BabA binds to Lewis B antigens and Le^b^ [33], SabA binding to sialyl-Le^x^ [34], while LPS binding occurs to Toll-like receptor 4 (TLR4) [35,36]. To date, there is no conclusive evidence on how *H. pylori* colonizes the biliary system. Recently, we reported that a mucinase-like enzyme is one of the most abundant proteins detected in *O. viverrini* excretory-secretory products [37]. This *O. viverrini* mucinase-like enzyme may degrade or modify the mucous barrier on the bile duct epithelium and facilitate *H. pylori* adhesion and colonization. To establish colonization in the biliary system, *H. pylori* adhesins must bind to their specific receptors on the biliary epithelium. It is well-known that cholangiocytes express a variety of pathogen-recognition receptors including TLRs, particularly TLR4 [38] and MUC5AC, in health and diseases [39], which are the receptors for *H. pylori* [40,41]. Given that *O. viverrini* can induce overexpression of TLR4 [42] and MUC5AC [43], the liver fluke may enhance colonization through these host receptors. 

Once *H. pylori* is colonized, it can activate cascades of signaling pathways leading to inflammatory cytokine release, cell proliferation, transformation, and malignancy [40,44]. Our study using an animal model now reports that *O. viverrini* and *H. pylori* co-infection enhances pathological and pre-neoplastic patterns, including goblet cell metaplasia, biliary hyperplasia, and dysplasia. Pre-cancerous lesions were detected at higher frequencies in the *O. viverrini* and *cag*A-positive *H. pylori* co-infection group than in any other group. In addition, hamsters that were co-infected with *O. viverrini* and *cag*A-positive *H. pylori* showed significantly reduced E-cadherin expression, especially in areas with dysplastic epithelium compared to *O. viverrini* infection alone or *O. viverrini* co-infected with *cag*A-negative *H. pylori*. The lower expression of E-cadherin in *O. viverrini* infection alone may be due to interleukin 6 (IL-6) and TGF- β1 production during infection [42,45]. IL-6 and TGF-β1 induced by excretory-secretory products reported in the human liver fluke, *Clonorchis sinensis* have been shown to down-regulate E-cadherin expression [46]. The more severe down-regulation of E-cadherin expression in the biliary epithelium of *O. viverrini* co-infected with *cag*A-positive *H. pylori* signifies the enhancement effect of the bacteria. CagA of *H. pylori* can downregulate E-cadherin expression and is involved in epithelial differentiation and transformation leading to malignancy [47]. Reduced levels of E-cadherin are commonly found in dysplastic tissue and pre-cancerous lesions, such as oral cancer [48,49], gastric cancer [50], and gallbladder cancer [51]. In CCA, the reduction in E-cadherin expression is associated with cell transformation, tumor progression, invasion, and metastasis [52]. Overall, our results are in agreement with previous studies on the CagA virulence factor and the higher severity of *H. pylori* infection, gastritis, and gastric carcinogenesis [53,54,55,56,57].

In summary, this study is the first to demonstrate that the carcinogenic liver fluke, *O. viverrine,* facilitates migration and colonization of *H. pylori* in the bile ducts. In addition, we showed that *O. viverrini* preferentially allows the *cag*A-positive strain of *H. pylori* to colonize in the gut and the tegument. The results support the view that *O. viverrini* is a reservoir host of *H. pylori*, particularly the *cag*A-positive strain. In turn, the fluke may continuously release the bacteria into the bile fluid. This phenomenon allows these two carcinogenic pathogens to establish a chronic co-infection. Consequently, chronic co-infection further enhances carcinogenic phenotypic changes of the bile duct epithelium leading to bile duct malignancy. This study provides fundamental information for further investigations regarding molecular pathways on carcinogenesis of *H. pylori* and liver fluke co-infection-associated bile duct cancer.

## Figures and Tables

**Table 1:** Detection of *H. pylori* in first order and second order bile ducts as well as worms using immunofluorescent staining. +++ = high numbers of *H. pylori* detection, ++ = moderate numbers of *H. pylori* detection, + = mild numbers of *H. pylori* detection. - = no *H. pylori* detection, NA = Not applicable

Month.	Groups	1st Order Bile Duct			2nd Order Bile Duct	Worm	
		Surface	Peri-Nucleus	Crypt		Tegument	Gut
1	OV/HP+	++	+	20% (1/5)	-	+	+
	OV/HP-	+++	+	0% (0/5)	-	+	-
	HP+	-	-	0% (0/5)	-	NA	NA
	HP-	-	-	0% (0/5)	-	NA	NA
3	OV/HP+	+	+++	20% (1/5)	-	+	+
	OV/HP-	+	+++	0% (0/5)	-	+	-
	HP+	-	-	0% (0/5)	-	NA	NA
	HP-	-	-	0% (0/5)	-	NA	NA

## Data Availability

Not applicable.
